# Editorialets

**Published:** 1906-10

**Authors:** 


### Editorialets.

The Worm Turns.—"You are another." The much-pounded, pelted, and persecuted ten-thousand-dollar-editor of the Octopus hits back. He pitches into the *New York Medical Record* with an energy that is surprising this hot weather, and wears him out "bodaciously"—an energy born of desperation and long-suffering. In the Octopus of September 15, the leading editorial is devoted to denouncing the *Medical Record*—some two or three pages being given to it. It fairly bristles with "false," "falsehood," "maliciously false," "mendacious," etc. It makes good reading these sweltering afternoons. "It is to laugh." "Go it, old woman, go it, bear!"

---

Changes in Asylum Officers.—Dr. L. T. Kirk, first assistant physician, and Dr. J. H. Eastland, second assistant, at the State Insane Asylum at Austin, resigned September 1st. Dr. Kirk will locate for general practice in Austin. Dr. Eastland will enter the Military Medical College at Washington, D. C., and take a two-years' course, when he will be assigned to the National Guard (Texas troops). His tuition and expenses will be paid by the U. S. Government, I understand. Dr. Eastland is connected with the State troops, being a regimental surgeon with rank of captain.

Their places at the asylum have been filled by appointment by the Governor upon recommendation of the superintendent and the board of trustees, by Dr. Margaret Holliday, of Austin, a graduate of the Medical Department of the University of Texas, and Dr. Horace Gilbert, who resigns the position of physician to the Confederate Home to accept the position mentioned. This leaves a vacancy in the office of physician to the Home, which, at the time of writing this, has not been filled. Dr. Gilbert resigned an asylum position to take that of physician to the Home, and now goes back. Dr. Holliday is the first woman physician to receive such appointment in Texas.

---

CREATE an active insurance committee in your county if you do not already possess one. The American Medical Association has such an active committee; the State Association now has one. Many county societies are already so organized. Examiners can only obtain just fees from the insurance companies by concerted action. The insurance lists in this issue will render correspondence easy; the arguments there suggested may be indefinitely amplified. Let every county society instruct its committee to at once begin an active correspondence with the medical directors, officers, trustees, State and local agents, remonstrating against the recent reductions, against an outside party dictating medical fees. Present the reasons of the medical profession for demanding living fees, and request an immediate return to the old rate, or the adoption of a new and reasonable schedule. Now for "a long pull, a strong pull and a pull all together."—*Texas State Journal of Medicine.*

[That is my platform. I endorse the position the Association has taken; and urge all my insurance-examiner readers to stand pat on a $5 minimum fee.—ED.]

---

THE TRI-STATE MEDICAL SOCIETY (Arkansas, Louisiana, and Texas) will meet at Marshall, Texas, November 14, 1906. Dues, $2. All members receive the *Medical Recorder,* of which Dr. Dowling, the president, is editor and proprietor (Shreveport, La.). The other officers are: Dr. C. M. Rosser, Dallas, first vice-president; Dr. M. G. Thompson, Hot Springs, Ark., second vice-president; Dr. S. A. Poole, Ruston, La., third vice-president; Dr. R. M. T. Mann, Texarkana, Ark., secretary.

---

DR. G. H. MOODY'S new half-page advertisement (front form) will interest you. His sanitarium is a beautiful spot, in the center of a large natural park in the suburbs, on beautiful Breckenridge avenue. The doctor is doing a splendid and successful business. He has recently made new additions to his buildings and equipments, among others a new and complete system for steam heating. See his pretty ad and write him for further details.

---

ORGANIZATION MEETING.—The Committee on Organization of the proposed "Medical Association of the Southwest," has called a meeting at Oklahoma City, for October 30 and 31, at which time joint sessions will be held with the Tri-State Medical Society of Texas, Oklahoma, and Arkansas. Dr. F. H. Clark, temporary secretary, El Reno, Okla.

---

BACK TO TEXAS.—Dr. Vard H. Hulen, who for some years practiced in Galveston, whence he removed to San Francisco seven years ago, has returned to Texas and located at Houston to practice his specialty, diseases of the eye. The profession of Texas with whom Dr. Hulen is deservedly popular, welcomes him back. The first thing he did was to renew his subscription to the "Red Back."

---

MISSISSIPPI VALLEY MEDICAL ASSOCIATION.—The next meeting of the Mississippi Valley Medical Association will be held at Hot Springs, Ark., November 6, 7 and 8, under the presidency of Dr. J. H. Carstens, of Detroit, Mich. The annual addresses will be delivered by Dr. Frank Parsons Norbury, Jacksonville, Ill., in medicine, and by Dr. Florus F. Lawrence, of Columbus, O., in surgery. Dr. Norbury has chosen for the subject of his address, "Clinical Psychology," and Dr. Lawrence will discuss in his address, "Surgical Principles and Theories." In addition to these addresses there will be the annual address of the president, Dr. Carstens. Elaborate arrangements have been made by the local profession of Hot Springs to entertain the visiting doctors and their wives, the meeting being held at the "Eastman" hotel, which will be specially opened in advance of the season to accommodate the association. A cordial invitation is extended to every physician in the valley to attend this meeting for which a large number of interesting and valuable papers have been promised. The headquarters will be at the beautiful "Arlington," where reduced rates will be in effect for the occasion. We would urge our readers to make early reservation of rooms, and avoid the risk of being crowded out, as the attendance is sure to be large. Communications regarding papers should be addressed to the secretary, Dr. Henry E. Tuley, 111 W. Kentucky street, Louisville, Ky.

---

TO JULY, 1911.—Dr. Isaac E. Clark, Schulenburg, Texas, sends $5, which pays his subscription to July, 1911. By that time Bryan will be President and Clark in Congress, I hope. He writes: "I want the 'Red Back' as long as I live and you edit it." He has been taking it now twenty-one consecutive years.

A GREAT DOCTOR.—By the bye, Clark incidentally tells me of a good joke on one of our most distinguished physicians, Dr. Mac.

He sent for Mac. to see a case in consultation with him. He, of course, introduced him to the proprietress of the hotel. She shook hands with him and said, "Doctor, I know you are one of the greatest doctors in the State." Mac. bowed and smole a smile, tickled to death. She went on: *"I see your advertisement in all the papers."* Phancy his pheelings! "No, my dear madam; I'm not that kind of a doctor," said Mac., blushing like a school boy. I'm going to tell it on him when I get him in a crowd.

